# Supplementary material for: EnzML: multi-label prediction of enzyme classes using InterPro signatures
Source: BMC Bioinformatics. 2012 Apr 25;13:61. doi: 10.1186/1471-2105-13-61 (PMC3483700; doi:10.1186/1471-2105-13-61)
Supplement: Addtional file 5 — The Java code to format the data files, evaluate and predict. The file enzml_java_code.tar.gz contains the Java code used to format database data to ARFF and XML formats, to execute cross and train-test (jackknife) evaluations and to record evaluation results to database. More information is included in the readme.txt file and the Javadoc files. The code can be used with a MySQL database. To use a different database software, other JDBC drivers might be required. [file 1471-2105-13-61-S5.gz › java_code/utils/doc/cern/jet/random/engine/RandomEngine.html]

RandomEngine


---


|  |  |  |
| --- | --- | --- |
| **Overview** \| **Package** \| **Class** \| **Use** \| **Tree** \| **Deprecated** \| **Index** \| **Help** | |  |
| **PREV CLASS**   NEXT CLASS | **FRAMES**    **NO FRAMES**     **All Classes** |  |
| SUMMARY: NESTED \| FIELD \| CONSTR \| METHOD | DETAIL: FIELD \| CONSTR \| METHOD |  |


---


## cern.jet.random.engine Class RandomEngine

```
java.lang.Object
  cern.colt.PersistentObject
      edu.cornell.lassp.houle.RngPack.RandomElement
          edu.cornell.lassp.houle.RngPack.RandomSeedable
              cern.jet.random.engine.RandomEngine
```

**All Implemented Interfaces:**: DoubleFunction, IntFunction, java.io.Serializable, java.lang.Cloneable

**Direct Known Subclasses:**: MersenneTwister

---

``` public abstract class RandomEngine extends RandomSeedable implements DoubleFunction, IntFunction ```

Abstract base class for uniform pseudo-random number generating engines.

Most probability distributions are obtained by using a **uniform**
pseudo-random number generation engine (derived from this class or
`RandomElement`) followed by a
transformation to the desired distribution. Thus, subclasses of this class
are at the core of computational statistics, simulations, Monte Carlo
methods, etc.

Subclasses produce uniformly distributed int's and long's
in the closed intervals [Integer.MIN\_VALUE,Integer.MAX\_VALUE] and
[Long.MIN\_VALUE,Long.MAX\_VALUE], respectively, as well as
float's and double's in the open unit intervals
(0.0f,1.0f) and (0.0,1.0), respectively.

Subclasses need to override one single method only: nextInt(). All
other methods generating different data types or ranges are usually layered
upon nextInt(). long's are formed by concatenating two 32
bit int's. float's are formed by dividing the interval
[0.0f,1.0f] into 2^32^ sub intervals, then randomly
choosing one subinterval. double's are formed by dividing the
interval [0.0,1.0] into 2^64^ sub intervals, then randomly
choosing one subinterval.

Note that this implementation is **not synchronized**.

**Version:**
:   1.0, 09/24/99

**Author:**
:   wolfgang.hoschek@cern.ch

**See Also:**: `MersenneTwister`, `Ranmar`, `Ranlux`, `Ranecu`, `Random`, Serialized Form

---

| **Method Summary** | |
| --- | --- |
| `double` | `apply(double dummy)`             Equivalent to raw(). |
| `int` | `apply(int dummy)`             Equivalent to nextInt(). |
| `static RandomElement` | `makeDefault()`             Constructs and returns a new uniform random number engine seeded with the current time. |
| `double` | `nextDouble()`             Returns a 64 bit uniformly distributed random number in the open unit interval `(0.0,1.0)` (excluding 0.0 and 1.0). |
| `float` | `nextFloat()`             Returns a 32 bit uniformly distributed random number in the open unit interval `(0.0f,1.0f)` (excluding 0.0f and 1.0f). |
| `abstract  int` | `nextInt()`             Returns a 32 bit uniformly distributed random number in the closed interval [Integer.MIN\_VALUE,Integer.MAX\_VALUE] (including Integer.MIN\_VALUE and Integer.MAX\_VALUE); |
| `long` | `nextLong()`             Returns a 64 bit uniformly distributed random number in the closed interval [Long.MIN\_VALUE,Long.MAX\_VALUE] (including Long.MIN\_VALUE and Long.MAX\_VALUE). |
| `double` | `raw()`             Returns a 32 bit uniformly distributed random number in the open unit interval `(0.0,1.0)` (excluding 0.0 and 1.0). |

**Methods inherited from class edu.cornell.lassp.houle.RngPack.RandomSeedable**

`ClockSeed, ClockSeed`

**Methods inherited from class edu.cornell.lassp.houle.RngPack.RandomElement**

`choose, choose, gaussian, gaussian, powlaw, raw, raw, uniform`

**Methods inherited from class cern.colt.PersistentObject**

`clone`

**Methods inherited from class java.lang.Object**

`equals, getClass, hashCode, notify, notifyAll, toString, wait, wait, wait`

**Method Detail**

### apply

```
public double apply(double dummy)
```

:   Equivalent to raw(). This has the effect that random engines can
    now be used as function objects, returning a random number upon function
    evaluation.

    :   **Specified by:**: `apply` in interface `DoubleFunction`
    :   **Parameters:**: `dummy` - argument passed to the function. **Returns:**: the result of the function.

---


### apply

```
public int apply(int dummy)
```

:   Equivalent to nextInt(). This has the effect that random engines
    can now be used as function objects, returning a random number upon
    function evaluation.

    :   **Specified by:**: `apply` in interface `IntFunction`
    :   **Parameters:**: `dummy` - argument passed to the function. **Returns:**: the result of the function.

---


### nextDouble

```
public double nextDouble()
```

:   Returns a 64 bit uniformly distributed random number in the open unit
    interval `(0.0,1.0)` (excluding 0.0 and 1.0).

---


### nextFloat

```
public float nextFloat()
```

:   Returns a 32 bit uniformly distributed random number in the open unit
    interval `(0.0f,1.0f)` (excluding 0.0f and 1.0f).

---


### nextInt

```
public abstract int nextInt()
```

:   Returns a 32 bit uniformly distributed random number in the closed
    interval [Integer.MIN\_VALUE,Integer.MAX\_VALUE] (including
    Integer.MIN\_VALUE and Integer.MAX\_VALUE);

---


### nextLong

```
public long nextLong()
```

:   Returns a 64 bit uniformly distributed random number in the closed
    interval [Long.MIN\_VALUE,Long.MAX\_VALUE] (including
    Long.MIN\_VALUE and Long.MAX\_VALUE).

---


### raw

```
public double raw()
```

:   Returns a 32 bit uniformly distributed random number in the open unit
    interval `(0.0,1.0)` (excluding 0.0 and 1.0).

    :   **Specified by:**: `raw` in class `RandomElement`
    :   **Returns:**: a random double in the range (0,1). **See Also:**: `RandomJava`

---


### makeDefault

```
public static RandomElement makeDefault()
```

:   Constructs and returns a new uniform random number engine seeded with the
    current time. Currently this is
    `MersenneTwister`.


---


|  |  |  |
| --- | --- | --- |
| **Overview** \| **Package** \| **Class** \| **Use** \| **Tree** \| **Deprecated** \| **Index** \| **Help** | |  |
| **PREV CLASS**   NEXT CLASS | **FRAMES**    **NO FRAMES**     **All Classes** |  |
| SUMMARY: NESTED \| FIELD \| CONSTR \| METHOD | DETAIL: FIELD \| CONSTR \| METHOD |  |


---
